# The Book World of Medicine and Science

**Published:** 1897-05-01

**Authors:** 


### THE BOOK WORLD OF MEDICINE AND SCIENCE.

Aids to the Study of Bacteriology. By T. H. Pearmain and C. G. Moor, M.A. (London: Ballière, Tindall, and Cox. Price 3s. 6d.)

For certain classes of readers this will be found a most useful handbook. It is not everyone who wishes to be a specialist, yet every student and practitioner of medicine requires to know somewhat of bacteriology, and for such this book gives much useful information in a most convenient form. A short sketch is given of the history of bacteriology, of the methods of reproduction, the classification, the conditions of growth of, and the degrees of resistance to outside influences possessed by the various kinds of micro-organisms. A sketch is given of the apparatus used and the various nutrient media employed in bacteriological research, and then comes a careful chapter on the methods by which the microbes are cultivated, examined, and differentiated. In a series of short chapters the chief pathogenic bacteria are then individually described, and a good deal of information is given in regard to fermentations, the products of the metabolism of organisms, and the bacteriological examination of air, water, and soil. It will be seen, then, that, although space prevents any part of the subject being very deeply gone into, the whole range of the science is touched upon so that the reader is able to obtain a very considerable insight into the mysteries of the science, and even some slight smattering of the jargon employed among themselves by its votaries, which latter is not entirely without its uses from the frequency with which such terms are used in general medical literature. Of its kind this little book is good, but when medical affairs are touched upon the authors appear somewhat out of their depth.

The Menopause and its Disorders. By A. D. Leith Napier, M.D., M.R.C.P. (London: The Scientific Press. 1897. Price 7s. 6d. net.)

There can be no doubt that there was room for a book on this subject. Anyone in ordinary mixed practice who will look back on the work of any single week will see at once how largely the diseases and the ailments of the menopause figure among the maladies with which he has to deal in his everyday life. It is true that most of these ailments are treated of in their proper places in works on general medicine, but the longer one lives the more sure one feels that the period of the menopause engrafts its own peculiarities upon even the most ordinary ailments, and that to such an extent that it may often become a question when treatment has to be considered, and perhaps even more so when prognosis is the matter under discussion, whether the time of life is not an even more important factor in the case than the particular disease from which the patient may be stated to be ailing. The first portion of the book before us is devoted to anatomical and physiological considerations, and to an enunciation of Dr. Leith Napier's views as to the cause of menstruation. Then comes a very good account of normal change of life, and of the many lesser variations which may be included under the term "normal," and after that comes the mass of the book devoted to the disorders attending the change of life. As to the time of age at which the change of life comes on an investigation of 700 showed that the average age for definite cessation was forty-seven years and eight months. Some of the strange tales which are current as to menstruation continuing and children being born at advanced periods of life are severely criticised. The author's own experience is to the effect that a considerable number of healthy women in all ranks of life do not reach the menopause till after their fiftieth year. "From fifty to fifty-four the number gradually decreases, and at fifty-five very few absolutely healthy women will be found menstruating." Cases in which so-called menstruation returns after a cessation are to be placed in a different category, and he says that "of 500 post-climacteric cases 36·5 per cent. had a return of hæmorrhage after the menopause had been established a year or more. Of these, cancer of the cervix caused the bleeding in 54 per cent. of the cases."

In dealing with the treatment of the various nervous diseases of the menopause the author protests against the routine use of bromides, which, varied by additions of either valerian, sumbul, assafœtida or musk, is regarded by so many as the sheet-anchor of treatment; and he points out how important it is that before giving bromides, by which symptoms are masked as well as relieved, we should restore as far as may be the disturbed functions which have increased, or even brought about, the nervous derangement.

A very useful chapter deals with the hæmorrhages of the menopause, special stress being laid upon the fact that excessive loss, especially true hæmorrhage, at this time is not to be looked upon, as is so often done, as natural to the climacteric period, and that in a large number of cases it arises from organic causes—prominent among which are the endometritis of the menopause and the development of new growths.

In regard to malignant disease, to which there is so special a proclivity during the fifth decennial period of life, emphasis is rightly laid on the importance of making an examination at the earliest possible moment whenever there is the slightest doubt or suspicion as to its presence. The early symptoms are often so slight, and when once ulceration has occurred progress is often so rapid, that cases which on being first seen might have presented favourable conditions for cure by operation may in two or three weeks pass beyond all hope.

The list of the diseases, not to mention the disorders, by which women are apt to be troubled at and about the menopause is so long that it is quite possible that certain omissions may be discovered in the book before us; nevertheless, Dr. Leith Napier is to be congratulated on the production of a volume which deals in a very interesting and suggestive manner with a most important group of topics—a volume which is well printed, well illustrated, and is likely to be of great practical use to the general practitioner as well as to those more specially engaged in gynæcological work.

### BOOKS RECEIVED.

Scientific Press.

